# Associations between QT interval subcomponents, HIV serostatus, and inflammation

**DOI:** 10.1111/anec.12705

**Published:** 2019-09-19

**Authors:** Katherine C. Wu, Fiona Bhondoekhan, Sabina A. Haberlen, Hiroshi Ashikaga, Todd T. Brown, Matthew J. Budoff, Gypsyamber D'Souza, Jared W. Magnani, Lawrence A. Kingsley, Frank J. Palella, Joseph B. Margolick, Otoniel Martínez‐Maza, Sean F. Altekruse, Elsayed Z. Soliman, Wendy S. Post

**Affiliations:** ^1^ Division of Cardiology Department of Medicine Johns Hopkins University School of Medicine Baltimore Maryland; ^2^ Department of Epidemiology Johns Hopkins Bloomberg School of Public Health Baltimore Maryland; ^3^ Division of Endocrinology, Diabetes and Metabolism Department of Medicine Johns Hopkins University School of Medicine Baltimore Maryland; ^4^ Los Angeles Biomedical Research Institute at Harbor‐UCLA Medical Center Los Angeles California; ^5^ School of Medicine University of Pittsburgh Medical Center University of Pittsburgh, and the Heart and Vascular Institute Pittsburgh Pennsylvania; ^6^ Departments of Infectious Diseases and Microbiology and Epidemiology University of Pittsburgh Graduate School of Public Health Pittsburgh Pennsylvania; ^7^ Department of Medicine Northwestern University Feinberg School of Medicine Chicago Illinois; ^8^ Department of Molecular Microbiology and Immunology Johns Hopkins Bloomberg School of Public Health Baltimore Maryland; ^9^ Department of Obstetrics and Gynecology, Microbiology, Immunology & Molecular Genetics David Geffen School of Medicine at UCLA UCLA Fielding School of Public Health Los Angeles California; ^10^ Department of Epidemiology UCLA Fielding School of Public Health Los Angeles California; ^11^ Epidemiology Branch, Prevention and Population Sciences Program Division of Cardiovascular Sciences National Heart, Lung, and Blood Institute Bethesda Maryland; ^12^ Cardiology Section Department of Epidemiology and Prevention and Department of Medicine Epidemiological Cardiology Research Center (EPICARE) Wake Forest School of Medicine Winston‐Salem North Carolina

**Keywords:** arrhythmias, electrocardiography, HIV, inflammation, QT interval

## Abstract

**Background:**

The total QT interval comprises both ventricular depolarization and repolarization currents. Understanding how HIV serostatus and other risk factors influence specific QT interval subcomponents could improve our mechanistic understanding of arrhythmias.

**Methods:**

Twelve‐lead electrocardiograms (ECGs) were acquired in 774 HIV‐infected (HIV+) and 652 HIV‐uninfected (HIV−) men from the Multicenter AIDS Cohort Study. Individual QT subcomponent intervals were analyzed: R‐onset to R‐peak, R‐peak to R‐end, JT segment, T‐onset to T‐peak, and T‐peak to T‐end. Using multivariable linear regressions, we investigated associations between HIV serostatus and covariates, including serum concentrations of inflammatory biomarkers such as interleukin‐6 (IL‐6), and each QT subcomponent.

**Results:**

After adjustment for demographics and risk factors, HIV+ versus HIV− men differed only in repolarization phase durations with longer T‐onset to T‐peak by 2.3 ms (95% CI 0–4.5, *p* < .05) and T‐peak to T‐end by 1.6 ms (95% CI 0.3–2.9, *p* < .05). Adjusting for inflammation attenuated the strength and significance of the relationship between HIV serostatus and repolarization. The highest tertile of IL‐6 was associated with a 7.3 ms (95% CI 3.2–11.5, *p* < .01) longer T‐onset to T‐peak. Age, race, body mass index, alcohol use, and left ventricular hypertrophy were each associated with up to 2.2–12.5 ms longer T‐wave subcomponents.

**Conclusions:**

HIV seropositivity, in combination with additional risk factors including increased systemic inflammation, is associated with longer T‐wave subcomponents. These findings could suggest mechanisms by which the ventricular repolarization phase is lengthened and thereby contribute to increased arrhythmic risk in men living with HIV.

## 1 INTRODUCTION

Lengthening of the electrocardiographic QT interval is associated with increased risk for sudden cardiac death (SCD) in the general population(Montanez, Ruskin, Hebert, Lamas, & Hennekens, 2004; Soliman et al., 2011; Straus et al., 2006) and atrial fibrillation in community‐based cohorts(Mandyam et al., 2013; O'Neal et al., 2015) via shared potassium and sodium currents that impact both ventricular and atrial repolarization (Nerbonne & Kass, 2005). As people with human immunodeficiency virus (HIV) live longer, they are at increased susceptibility for cardiac arrhythmias and SCD (Hsu et al., 2013; Tseng et al., 2012) for which mechanisms are poorly understood. We recently reported an HIV‐associated lengthening of the corrected QT interval (QTc) not explained by HIV disease‐related factors but which was attenuated by higher concentrations of inflammatory biomarkers (Wu et al., 2019). While the QT interval in totality is used as a marker of ventricular repolarization, it is in fact comprised of both depolarizing and repolarizing current flows, specifically phases 0–3 of the ventricular action potential (Antzelevitch, 2007; Prenner, Shah, Goldberger, & Sauer, 2016; Yan, Lankipalli, Burke, Musco, & Kowey, 2003). The initial subcomponent of the QT consists of the QRS interval, signifying His–Purkinje conduction, myocardial depolarization, and an early component of repolarization. The second subcomponent, the JT interval, is a purer measure of myocardial repolarization and includes three segments: the ST segment and two T‐wave subcomponents, T‐wave onset to T‐wave peak, and T‐wave peak to T‐wave end. Longer T‐wave subcomponents have been associated with atrial and ventricular arrhythmias and SCD in HIV‐uninfected (HIV−) cohorts (Goldenberg & Moss, 2017; Morin et al., 2012; O'Neal et al., 2017; Panikkath et al., 2011; Roberts et al., 2017; Rosenthal et al., 2015; Shimizu et al., 2002; Yamaguchi et al., 2003). Whether these associations occur in HIV‐infected (HIV+) persons is unknown. In this study, we extended our QTc interval findings to investigate the relationship between specific QT subcomponent durations and HIV serostatus in our previously described large cohort of HIV+ and HIV− men who have sex with men (MSM). We additionally explored how the association between HIV infection and QT subcomponents was affected by other risk factors such as levels of inflammation.

## 2 METHODS

The study cohort comprised 1,612 active enrollees in the Multicenter AIDS Cohort Study (MACS) described previously (Wu et al., 2019). The MACS is a prospective, longitudinal study of MSM from four U.S. sites (Baltimore/Washington DC; Chicago, IL; Pittsburgh, PA and Los Angeles, CA) and which had four enrollment waves: 1984–1985, 1987–1991, 2001–2003, and 2010–2018. Both HIV+ and HIV− men are included who undergo semiannual visits during which standardized interviews, physical examinations and blood and urine specimen collection and assays are performed. Participants with a history of myocardial infarction, stroke, or heart failure (*n* = 49) were excluded from the present analyses.

Data collected included demographics (age, race), measured blood pressure, body mass index (BMI), smoking status, prescribed medications, recreational drug, and alcohol use, and laboratory values (fasting serum glucose and lipid levels and estimated glomerular filtration rate, eGFR). Medications were categorized by QT prolongation risk using the database CredibleMeds.org (Woosley, Heise, & Romero; Wu et al., 2019), accessed 12/14/17. Hepatitis C viral status was assessed by antibody and RNA testing (chronic infection defined as antibody‐ and RNA‐positive). In HIV+ men, measures of HIV disease activity included plasma HIV RNA concentrations (quantified down to 50 copies/ml, Roche ultrasensitive assay), current and nadir CD4+ T‐lymphocyte cell counts/ml, prior AIDS‐defining malignancy or opportunistic infection and antiretroviral therapy (ART) including use and duration of highly active ART (HAART) and protease inhibitors (PI).

Standard resting twelve‐lead electrocardiograms (ECGs) were performed between October 1, 2016, and October 1, 2017. All sites were trained extensively for optimal electrode positioning, room condition, data collection, and transfer. Digital ECGs were recorded at 10 mm/mV calibration at a speed of 25 mm/s for 10 s using General Electric (GE) MAC 1,600 ECG machines (Marquette Electronics) and transmitted for centralized reading to the ECG Reading Center at the Epidemiological Cardiology Research Center (EPICARE), Wake Forest University (Winston‐Salem, NC). Tracings were excluded (*n* = 137) from analysis because of poor quality (*n* = 32), nonsinus rhythm (e.g., atrial fibrillation/flutter, *n* = 15 or paced atrial rhythm, *n* = 5), or major intraventricular conduction defects (QRS duration ≥120 ms, Wolff–Parkinson–White syndrome, or ventricular pacemaker, *n* = 85). Left ventricular hypertrophy (LVH) was defined by the Cornell criteria (O'Neal et al., 2017).

Electrocardiogram parameters were automatically processed using the GE Marquette 12‐SL program (GE Marquette, Milwaukee, WI). As previously described, (Wu et al., 2019) for each tracing, a single global measure of the QT interval was defined as the time duration between the QRS onset to the latest T‐wave offset (end). The total QT interval duration was corrected for heart rate using the linear Framingham formula to obtain the QTc. To derive the individual QT interval component lengths, the median value in all 12 leads for each of the following was computed: intrinsicoid R wave (onset of R wave to R‐wave peak), R‐peak to R‐end (R‐wave peak to R‐wave end), JT segment (J‐point to T‐wave onset), T‐onset to T‐peak (T‐wave onset to T‐wave peak), and T‐peak to T‐end (T‐wave peak to T‐wave end) (O'Neal et al., 2017; Roberts et al., 2017). Due to the automated processing of the digitally acquired data, there are 0 variability and high repeatability (Gellert et al., 2014; Soliman, Prineas, Case, Zhang, & Goff, 2009).

Among enrollees who also participated in ancillary MACS studies [a study of subclinical atherosclerosis by computed tomography (Ketlogetswe et al., 2015) and a study of inflammation and immune activation biomarkers (Wada et al., 2015)], stored serum was analyzed for concentrations of seventeen biomarkers as described (Wu et al., 2019). We focused the current analysis on the three serum biomarkers associated with prolonged QTc in our prior paper: interleukin‐6 (IL‐6); B‐cell activation factor (BAFF); and intercellular adhesion molecule 1 (ICAM‐1) (Wu et al., 2019).

The Institutional Review Boards of all MACS sites approved the study, and all participants signed informed consent.

We report continuous data as mean values ± standard deviations (*SD*) or median values with interquartile ranges (IQRs). Distributions of the QT interval subcomponents by HIV serostatus were compared using Kolmogorov–Smirnov testing. Multivariable linear regressions were used to evaluate the associations between HIV serostatus and individual QT interval subcomponents. Missing covariate data (8%) were imputed using the Markov chain Monte Carlo procedure (*n* = 5).

We analyzed differences in the QT subcomponent durations with the following sequential models:

- *Model 1* included HIV serostatus only.
- *Model 2*: further adjusted for age, race, MACS site, year of MACS enrollment (before/after 2001), and ECG heart rate.
- *Model 3:* additionally adjusted for BMI, cumulative pack‐years of smoking, heavy alcohol use >13 drinks/week, systolic blood pressure, use of medications to treat hypertension or diabetes, opioid use, cocaine use, fasting glucose level, eGFR, presence of ECG LVH, and use of QT prolongation drugs (known + possible vs. conditional + none).

We also performed multivariable linear regressions in the HIV+ men to explore HIV‐associated disease factors including PI use, HIV RNA (viral load) level (< vs. ≥50 copies/ml), history of clinically defined AIDS, current and nadir CD4+ counts and HAART duration, each in a separate model including covariates from model 3. Among men with available inflammatory biomarker concentrations, we investigated the contribution of increasing tertiles of inflammatory biomarker levels (IL‐6, ICAM‐1, and BAFF) on the HIV association with the QT subcomponents, each in a separate model including covariates from model 3.

Further covariate adjustment for major electrocardiographic Q‐wave and ST‐T‐wave abnormalities, seen in a very small minority of individuals, did not change the results of our multivariable models and thus were not included in the final models.

Statistical significance was defined as *p*‐values < .05. All analyses were performed using SAS V.9.4.

## 3 RESULTS

The final sample size was *n* = 1,426 after exclusions. Table S1 summarizes the baseline, unadjusted cohort characteristics according to HIV serostatus. Compared to HIV− men, HIV+ men were older, more racially diverse, and had higher levels of inflammation. Most HIV+ men were virally suppressed (83%) and had received HAART for a median of 12.1 years. Table 1 summarizes unadjusted differences in the ECG parameters, while Table 2 reports mean differences in the QT subcomponent interval lengths for HIV+ versus HIV− men, adjusted for covariates. After adjustment that takes into account the baseline cohort differences, HIV serostatus was significantly associated with the T‐wave subcomponents of the QT interval (T‐onset to T‐peak and T‐peak to T‐end) but not with R‐onset to R‐peak, R‐peak to R‐end, or JT segment durations. The estimated HIV effect was similar between T‐onset to T‐peak (2.3 ms, 95% CI 0–4.5, *p* < .05) and T‐peak to T‐end (1.6 ms, 95% CI 0.3–2.9, *p* < .05).

**Table 1:** ECG parameters by HIV serostatus (unadjusted)

	Mean (*SD*), Median (IQR), or Number (%)		*p*‐value
	HIV− (*n* = 774)	HIV+ (*n* = 652)	
Heart rate (bpm)	64 (56,72)	67 (59,74)	**<.01**
QRS duration (ms)	90 (83.5,96)	88 (84,96)	.23
LVH (*n*) (%)	7 (1)	12 (2)	.43
QT duration (uncorrected) (ms)	403.6 (29.9)	399.1 (29.4)	**<.01**
QTc duration (Framingham) (ms)	411.3 (20)	412.1 (19.2)	.47
QTc clinical threshold > 450 ms (*n*) (%)	17 (3)	24 (3)	.58
QT subcomponent duration (ms) (*SD*)			
R‐onset to R‐peak	26.7 (5.6)	26.5 (5.2)	.89
R‐peak to R‐end	20.3 (7.8)	21.4 (9)	.21
JT segment	117.9 (20.2)	113.6 (20)	**<.01**
T‐onset to T‐peak	96.2 (20.5)	95.8 (19.9)	.56
T‐peak to T‐end	98.3 (11.8)	99.2 (11.9)	.30
JT interval (J‐point to T‐end)	313.3 (29.5)	309.4 (28.9)	**<.01**
T wave	195.5 (20.3)	196 (19.9)	.70

Significant p‐values, *p* < 0.05 are bolded.

**Table 2:** Adjusted^a^ mean difference in QT interval subcomponent durations (ms) by HIV serostatus

	Mean difference (95% CI)
R‐onset to R‐peak	0.0 (−0.6, 0.6)
R‐peak to R‐end	0.4 (−0.5, 1.4)
JT segment	−0.5 (−1.1, 0.0)
T‐onset to T‐peak	**2.3 (0.0, 4.5)** *
T‐peak to T‐end	**1.6 (0.3, 2.9)** *
Total QTc interval (Framingham correction)	**3.8 (1.7, 6.0)** **

a Each QT interval subcomponent was assessed in a separate model with adjustment for covariates included in Model 3, see text in Section 2 and Table 3 below.

Significant p‐values, *p* < 0.05 are bolded.

* *p* ≤ .05.

** *p* < .01 HIV+ compared to HIV−.

Table 3 shows the associations between HIV serostatus and T‐onset to T‐peak and T‐peak to T‐end, each after full adjustment. Tables S2 and S3 show the development of Models 1–3 for each T‐wave subcomponent. In addition to positive HIV serostatus, older age and heavy alcohol use were significantly associated with longer T‐onset to T‐peak. Covariates in addition to positive HIV serostatus associated with longer T‐peak to T‐end included age, black race, BMI, eGFR, and LVH. Higher heart rates correlated with shorter T‐peak to T‐end, as did higher eGFR. Older age was associated with a longer T‐onset to T‐peak (2.2 ms per 5‐year increments) compared to T‐peak to T‐end (0.4 ms per 5‐year increment).

**Table 3:** Risk factors for longer T‐onset to T‐peak and T‐peak to T‐end among all 1,426 participants in fully adjusted analyses (Model 3^a^)

	T‐onset to T‐peak^a^	T‐peak to T‐end^a^
	Mean difference (95% CI)	Mean difference (95% CI)
Intercept^b^	**94.5 (91.6, 97.5)** **	**97.1 (95.4, 98.9)** **
HIV‐infected (vs. uninfected)	**2.3 (0.0, 4.5)** *	**1.6 (0.3, 2.9)** *
Age per 5 years	**2.2 (1.6, 2.9)** **	**0.4 (0.1, 0.8)** *
Race		
Black (vs. Caucasian)	−1.6 (−4.5, 1.3)	**2.1 (0.4, 3.7)** *
Hispanic/Other (vs. Caucasian)	0.0 (−3.5, 3.5)	1.9 (−0.1, 3.9)
Enrolled after 2001	2.4 (−0.5, 5.4)	0.1 (−1.6, 1.9)
Heart rate per 5 bpm	0.3 (−0.2, 0.7)	**−1.1 (−1.4, −0.8)** **
BMI per 5 kg/m^2^	0.1 (−1.0, 1.2)	**1.5 (0.9, 2.1)** **
Alcohol use > 13 drinks per week	**5.8 (2.0, 9.6)** **	−0.8 (−3.0, 1.4)
Smoking (per 10 cumulative pack‐years)	0.5 (0.1, 1.0)	**−0.5 (−0.8, −0.2)** **
Opioid use	0.2 (−3.7, 4.2)	1.0 (−1.3, 3.4)
Systolic blood pressure per 10 mmHg	0.7 (0.0, 1.4)	0.0 (−0.4, 0.4)
Fasting glucose per 10 mg/dl	−0.1 (−0.5, 0.3)	0.0 (−0.3, 0.2)
On hypertension medications	1.1 (−1.3, 3.5)	0.1 (−1.3, 1.5)
On diabetes medications	0.6 (−3.6, 4.7)	−2.3 (−4.7, 0.1)
eGFR per 5 ml/min/1.73 m^2^	0.1 (−0.2, 0.5)	**−0.2 (−0.4, 0.0)** *
LVH	−6.1 (−15, 2.9)	**12.5 (7.3, 17.8)** **
QT prolongation drugs (known + possible vs. conditional + none)	0.0 (−3.5, 3.5)	1.2 (−0.8, 3.3)
Cocaine use	3.5 (−0.3, 7.2)	0.2 (−2.0, 2.4)

a Model 3 further controlled for MACS enrollment site.

b Mean T‐onset to T‐peak and T‐peak to T‐end duration among men at the average and referent values of continuous and categorical covariates (see Table S2 footer for details).

Significant p‐values, *p* < 0.05 are bolded.

* *p* ≤ .05

** *p* < .01.

Among HIV+ men, few markers of HIV disease activity or ART were related to T‐wave subcomponents (Table S4). PI use was associated with a 3.3 ms (95% CI 0–6.5) *shorter* T‐onset to T‐peak and a 3.1 ms (95% CI 1.1–5.0) *longer* T‐peak to T‐end with a net neutral effect on the overall T wave. Having an undetectable HIV RNA level (vs. >50 copies/ml) was associated with a 2.9 ms (95% CI 0.6–5.1) shorter T‐peak to T‐end but no significant relationship with T‐onset to T‐end. The HIV effect on the T‐wave subcomponents was similar when we limited the analyses to men who were virally suppressed and had no history of AIDS (2.4 ms difference for T‐onset to T‐peak, 95% CI 0–4.8 and 1.4 ms difference for T‐peak to T‐end, 95% CI 0.1–2.8).

Increasing tertile levels of IL‐6, ICAM‐1, and BAFF attenuated the strength and significance of the HIV association between T‐onset and T‐peak and T‐peak to T‐end. Men with the highest compared to lowest inflammatory biomarker concentrations had a 4.2–7.3 ms longer T‐onset to T‐peak (Table 4).

**Table 4:** Adjusted associations between HIV infection, inflammation, and longer T‐onset to T‐peak and T‐peak to T‐end durations among men with all three available inflammatory biomarker testing results (*n* = 574 men, 230 HIV−, and 344 HIV+)

	Mean difference in T‐onset to T‐peak (ms, 95% CI)				Mean difference in T‐peak to T‐end (ms, 95% CI)			
	Adjusted Model^a^	IL‐6^a^	ICAM‐1^a^	BAFF^a^	Adjusted Model^a^	IL‐6^a^	ICAM‐1^a^	BAFF^a^
HIV infection (vs. uninfected)	**2.3 (0.0, 4.5)** *	1.6 (−2.1, 5.3)	1.3 (−2.5, 5.0)	1.6 (−2.2, 5.4)	**1.6 (0.3, 2.9)** *	0.7 (−1.6, 3.0)	0.6 (−1.7, 2.9)	0.2 (−2.1, 2.5)
Tertile								
1	–	Ref	Ref	Ref	–	Ref	Ref	Ref
2	–	**6.9 (2.9, 11.0)** **	**4.0 (0.0, 8.1)** *	1.7 (−2.4, 5.8)	–	−1.1 (−3.6, 1.5)	0.1 (−2.4, 2.6)	0.3 (−2.2, 2.8)
3	–	**7.3 (3.2, 11.5)** **	**6.0 (1.7, 10.4)** **	**4.2 (−0.1, 8.5)** *	–	−0.4 (−2.9, 2.2)	0.3 (−2.4, 2.9)	2.2 (−0.4, 4.8)

a Each model included all variables specified in the fully adjusted model (Model 3), listed in Table 3.

Significant p‐values, *p* < 0.05 are bolded.

* *p* ≤ .05.

** *p* < .01.

## 4 DISCUSSION

A major strength of this study lies in the prevalent cohort design with concurrently enrolled HIV+ and HIV− men with similar lifestyles who were very well characterized. These features uniquely allow us to evaluate HIV effects in an unbiased manner and isolate differences among those HIV+ men who were virally suppressed and without a history of clinically defined AIDS, consistent with most contemporary HIV+ persons receiving care in the United States.

In this study, we extend our prior findings of an HIV‐associated increase in total QTc interval by reporting here that there is specific lengthening of the ventricular repolarization phase, as reflected by longer T‐wave subcomponent durations without significant changes in the other QT subcomponents. These results support the possibility of direct HIV effects on the hERG K+ channels which are active during ventricular repolarization. Higher levels of systemic inflammation contributed to the observed association between HIV+ serostatus and T‐onset to T‐peak wave lengthening. There were differences in the covariates that correlated with each T‐wave subcomponent. Although the degree of T‐wave subcomponent prolongation was small for each covariate, there is no standard threshold for clinical significance and the relationship between QT prolongation and outcomes has been shown to be linearly related (O'Neal et al., 2017; Panikkath et al., 2011). Moreover, individuals with multiple risk factors could have significant lengthening of their T‐wave intervals, supporting the “multi‐hit theory” whereby multiple insults lead to clinically significant QT prolongation (Lazzerini, Capecchi, El‐Sherif, Laghi‐Pasini, & Boutjdir, 2018). All of these findings corroborate the concept of marked heterogeneity within the QT interval and the importance of focusing on the repolarization phase by isolating differences in individual T‐wave subcomponent durations rather than the total QT interval.

T‐onset to T‐peak duration occurs during the early component of phase 3 of the action potential during which the rapid and slow components of the delayed rectifier potassium current operate (Yan et al., 2003). T‐peak to T‐end represents the transmural dispersion of repolarization and denotes the time when ventricular cells are vulnerable to early afterdepolarizations and ventricular arrhythmias (Antzelevitch, 2007; Prenner et al., 2016; Yan et al., 2003). Prior studies have reported the association between longer T‐peak to T‐end and increased risk for SCD in cohorts with pre‐existing heart disease (Goldenberg & Moss, 2017; Morin et al., 2012; Panikkath et al., 2011; Rosenthal et al., 2015; Shimizu et al., 2002; Yamaguchi et al., 2003). In contrast, prolonged T‐onset to T‐peak is associated with increased SCD risk and incident atrial fibrillation in community‐based cohorts without prevalent cardiovascular disease (O'Neal et al., 2017; Roberts et al., 2017). We find here a distinctive positive association between HIV infection and both T‐wave components. This could suggest viral effects on both potassium channels and transmural dispersion of repolarization and provide a unique rationale for the increased arrhythmic susceptibility in HIV+ people, particularly in conjunction with multiple other risk factors.

Notably, few indices of HIV‐related disease severity or usage of ART medications were associated with the T‐wave subcomponents. While PI use correlated with a longer T‐peak to T‐end, this was counteracted by a shorter T‐onset to T‐peak and explains our previously reported neutral effect on the total QTc (Wu et al., 2019). HIV viral suppression was associated with shorter T‐peak to T‐end, which supports an HIV effect on the transmural dispersion gradient, which warrants further study. The paucity of HIV‐related disease factor relationships with the T‐wave subcomponents raised the possibility of contributions from other factors, of which inflammation was a leading contender based on our prior work pertaining to the total QTc (Wu et al., 2019). Attenuation of the HIV association with T‐onset to T‐peak by higher inflammatory levels supports a potential mechanistic role of cytokine‐induced potassium channelopathies suggested by findings from cohort studies and supported by recent experimental studies (Aromolaran et al., 2018; Capecchi et al., 2019; Lazzerini, Laghi‐Pasini, Boutjdir, & Capecchi, 2019). Among patients with systemic inflammatory diseases but who are HIV−, QTc prolongation correlates with higher IL‐6 concentrations (Capecchi et al., 2019). Moreover, in people with rheumatoid arthritis, IL‐6 inhibitor therapy results in rapid QTc shortening in 3–6 months (Capecchi et al., 2019; Kobayashi et al., 2018; Lazzerini et al., 2015). Also recently described were 40 unselected patients with unexplained torsade de pointes in whom IL‐6 concentrations were markedly elevated at levels equivalent to those with severe rheumatoid arthritis (Lazzerini et al., 2017). In an in vitro study of guinea pig ventricular cardiomyocytes using electrophysiological and biochemical assays, IL‐6 suppressed the delayed rectifier potassium current and prolonged the action potential duration (Aromolaran et al., 2018). Our findings show the strongest association between higher IL‐6 levels and T‐onset to T‐peak with a 7.3 ms (95% 3.2, 11.5) longer duration among men with the highest tertile levels of IL‐6 which is consistent with an inflammatory potassium channelopathy mechanism whereby the hERG K + channel is a specific target for IL‐6.

Our results provide further insights into previously reported associations between the total QTc and covariates that may co‐occur in HIV+ men by underscoring differences in T‐wave subcomponent durations by HIV serostatus. Increases in total QTc with age have been reported(Rabkin, Cheng, & Thompson, 2016) and partly attributed to age‐related decreases in testosterone levels affecting calcium and potassium channels (van Noord, Rodenburg, & Stricker, 2011) Our finding of a stronger association between age and T‐onset to T‐peak than T‐peak to T‐end supports potassium channel‐related effects. The relationship we found between heavy alcohol use and T‐onset to T‐peak confirms an alcohol‐related correlation with prolonged QTc observed in a large HIV−, general cohort (Li et al., 2016). Experimentally, alcohol inhibits the repolarizing potassium currents carried by the hERG channels (namely the delayed rectifier current, I_Kr_, and the transient outward current, I_to_) (Bebarova, Horakova, & Kula, 2017) which could explain our results.

In contrast, dominant effects on T‐peak to T‐end support non‐channel‐related mechanisms. We found black race to be associated with T‐peak to T‐end which adds to prior reports of increased total QTc in blacks with coronary artery disease and SCD (Reinier et al., 2015; Williams et al., 2012). The association between increased BMI and T‐peak to T‐end suggests that greater dispersion of repolarization may partly explain why overweight and obese people have longer QTc which can shorten with weight loss (Omran, Bostick, Chan, & Alpert, 2018). Intracellular recordings from a rat model of LVH demonstrated enhanced transmural repolarization dispersion(Yan et al., 2001) which provides a rationale for LVH‐associated lengthening of T‐peak to T‐end seen here and increased mortality in persons with concomitant LVH and QTc prolongation compared to individuals without (Haugaa et al., 2014).

Limitations of our study include the inclusion of only men who were MSM. Our findings cannot be extrapolated to women. We excluded individuals with myocardial infarction, stroke and heart failure and extensively controlled for cardiac risk factors. Nonetheless, we are unable to completely control for subclinical coronary atherosclerosis, though adjusting for electrocardiographic evidence of major Q waves or abnormal ST‐T‐wave changes seen a very small number of participants did not change the results of our multivariable models. There remains the potential for bias from unknown confounders associated with HIV infection and the QT subcomponents. We cannot completely separate HIV effects from those of ART medications. While inflammatory biomarker levels were not measured concurrently with the ECG, their long‐term stability has been shown after HAART‐induced HIV viral suppression is achieved (Wada et al., 2015). Biomarker levels were only available in a subset of men. The present study explored potential hypotheses for the associations between HIV infection, inflammation, and T‐wave subcomponents; our study cannot prove causation. While ECG measurement error cannot be completely ruled out, any such error would be expected to affect the HIV cohorts equally and favor the null hypothesis.

In conclusion, the QTc prolongation observed in HIV+ men is associated with lengthening of the pure repolarization phase and both T‐wave subcomponents. People with greater systemic inflammation may have increased risk for abnormal repolarization, especially if other risk factors such as older age, black race, obesity, LVH, and/or heavy alcohol use co‐exist. Prolongation of QT subcomponent durations may be further potentiated by additional QT‐prolonging effects associated with HIV therapy that could merit increased vigilance and ECG surveillance. This study highlights the potential value of examining QT interval subcomponent durations rather than the total QT interval as a marker of risk and suggests possible mechanisms whereby longer QTc increases arrhythmic risk in people living with HIV.

## CONFLICT OF INTEREST

FJP is on the speaker's bureau for Gilead Sciences, Janssen Pharmaceuticals, Merck & Co., Inc., and Bristol‐Myers Squibb. MJB has received grants from General Electric Company. TTB has served as a consultant to Gilead Sciences, Merck, ViiV Healthcare, Theratechnologies, and EMD‐Serono. The other authors report no conflicts of interest.

## DISCLAIMER

The contents of this publication are solely the responsibility of the authors and do not represent the official views of the National Institutes of Health (NIH), NHLBI, U.S. Department of Health and Human Services, JHU ICTR, or NCATS.
