# Supplementary material for: Associations between QT interval subcomponents, HIV serostatus, and inflammation
Source: Ann Noninvasive Electrocardiol. 2019 Sep 19;25(2):e12705. doi: 10.1111/anec.12705 (PMC7358816; doi:10.1111/anec.12705)
Supplement: Supplementary file 1 [file ANEC-25-e12705-s001.docx]

| **Supplemental Table 1:** Characteristics of the study cohort by HIV-serostatus | | | |
| --- | --- | --- | --- |
|  | **N (%)** | |  |
|  | **HIV-uninfected (n=774)**  **(N=652)** | **HIV-infected (n=652)**  **(N=774)** | **p-value** |
| Age in years; median (IQR) | 61 (54.4,67.2) | 54.4 (46.9,60.6) | **<0.01** |
| Race (%) |  |  | **<0.01** |
| Caucasian | 471 (72) | 370 (48) |  |
| Black | 118 (18) | 255 (33) |  |
| Hispanic/Other | 63 (10) | 149 (19) |  |
| MACS site |  |  | **0.01** |
| Baltimore, MD | 154 (24) | 187 (24) |  |
| Chicago, IL | 104 (16) | 172 (22) |  |
| Pittsburgh, PA | 191 (29) | 188 (24) |  |
| Los Angeles, CA | 203 (31) | 227 (29) |  |
| Enrolled after 2001 | 232 (36) | 500 (65) | **<0.01** |
| Education level (below 12 years) | 91 (14) | 197 (25) | **<0.01** |
| Cumulative pack-year of smoking | 0.2 (0,17.4) | 2.0 (0,19.5) | 0.15 |
| Alcohol use >13 drinks per week | 63 (10) | 55 (7) | 0.08 |
| Body mass index (kg/m^2^); median (IQR) | 26.6 (23.8,30) | 25.9 (23.2,29.1) | **<0.01** |
| Systolic blood pressure (mmHg); median (IQR) | 130.5 (120,141) | 128 (119,138) | **<0.01** |
| On hypertension medications | 242 (38) | 255 (33) | 0.09 |
| On diabetes medications | 59 (9) | 72 (9) | 0.89 |
| On cholesterol lowering medications | 225 (35) | 265 (35) | 0.87 |
| Opioid use | 42 (6) | 61 (8) | 0.30 |
| Cocaine use | 42 (7) | 90 (12) | **<0.01** |
| Fasting glucose (mg/dL); median (IQR) | 94 (87,103) | 94 (87,103) | 0.69 |
| Total cholesterol (ml/dL); median (IQR) | 179 (157,203) | 176 (149,200) | **0.03** |
| HDL cholesterol (ml/dL); median (IQR) | 50.4 (42.9,60) | 46.4 (40,56) | **<0.01** |
| eGFR below 60 ml/min/m^2^ | 46 (7) | 85 (11) | **0.01** |
| Chronic HCV infection | 24 (4) | 50 (7) | **0.01** |
| QT prolongation drugs (known + possible) | 46 (7) | 93 (12) | **<0.01** |
| **Biomarkers; median (IQR)** |  |  |  |
| IL-6 (pg/mL, N=250 HIV- / 385 HIV+) | 1.2 (0.8,2) | 1.5 (1,2.3) | **<0.01** |
| BAFF (ng/mL, N= 580 HIV- / 637 HIV+) | 2.5 (2.2,2.9) | 2.7 (2.2,3.2) | **<0.01** |
| ICAM-1 (ng/mL, n=255 HIV–/388 HIV+) | 225 (192–265) | 259 (209–314) | **<0.01** |
| **HIV factors** | - |  |  |
| HIV RNA (viral load), <50 copies/ml | - | 636 (83) |  |
| HIV RNA copy number per mL if detectable;  median (IQR) | - | 318 (86,6639) |  |
| CD4+ T-cell count (cells/mm^3^) | - | 698 (521,884) |  |
| Nadir CD4+ T-cell count (cells/mm^3^);  median (IQR) | - | 335 (216,467) |  |
| On HAART | - | 698 (91) |  |
| Duration of HAART (years); median (IQR) | - | 12.1 (4.4,16.4) |  |
| On protease inhibitor (PI) | - | 183 (24) |  |
| Duration of PI use (years); median (IQR) | - | 3.7 (0,11.4) |  |
| On efavirenz | - | 21 (3) |  |
| On rilpivirine | - | 8 (1) |  |
| History of AIDS |  | 74 (10) |  |
| **ECG parameters** |  |  |  |
| Heart rate (bpm); median (IQR) | 64 (56–72) | 67 (59–74) | **<0.001** |
| QRS duration (ms); median (IQR) | 90 (83.5–96) | 88 (84–96) | 0.23 |
| LVH by Cornell voltage | 7 (1) | 12 (2) | 0.43 |
| Major Q-wave abnormality | 11 (2) | 5 (1) | 0.06 |
| Major ST-T wave abnormality | 28 (4) | 25 (3) | 0.29 |
| QTc duration (Framingham) (ms) (SD) | 411.3 (20) | 412.1 (19.2) | 0.47 |
| QTc duration (Bazett) (ms) (SD) | 416.7 (23.9) | 419.8 (23.9) | **0.02** |
| QTc duration (Hodges) (ms) (SD) | 412.6 (19.9) | 412.4 (19.4) | 0.92 |

| **Supplemental Table 2**: Risk factors for longer T-onset to T-peak among all 1426 participants, in unadjusted, univariate (model 1), partially adjusted (model 2) and fully adjusted (model 3) analyses. | | | |
| --- | --- | --- | --- |
|  | **Model 1** | **Model 2^§^** | **Model 3^§^** |
|  | Mean difference (95% CI) | Mean difference  (95% CI) | Mean difference  (95% CI) |
| Intercept^†^ | **96.2 (94.6, 97.7)**** | **95.3 (92.5, 98.0)**** | **94.5 (91.6, 97.5)**** |
| HIV-infected (vs. uninfected) | -0.3 (-2.4, 1.8) | 2.0 (-0.1, 4.2) | **2.3 (0.0, 4.5)*** |
| Age per 5 years |  | **2.4 (1.9, 3.0)**** | **2.2 (1.6, 2.9)**** |
| Race |  |  |  |
| Black (vs. Caucasian) |  | -1.0 (-3.8, 1.8) | -1.6 (-4.5, 1.3) |
| Hispanic/Other (vs. Caucasian) |  | -0.1 (-3.5, 3.4) | 0.0 (-3.5, 3.5) |
| Enrolled after 2001 |  | **2.9 (0.0, 5.9)*** | 2.4 (-0.5, 5.4) |
| Heart rate per 5 bpm |  | 0.4 (-0.1, 0.8) | 0.3 (-0.2, 0.7) |
| BMI per 5 kg/m^2^ |  |  | 0.1 (-1.0, 1.2) |
| Alcohol use >13 drinks per week |  |  | **5.8 (2.0, 9.6)**** |
| Smoking (per 10 cumulative pack-years) |  |  | 0.5 (-0.1, 1.0) |
| Opioid use |  |  | 0.2 (-3.7, 4.2) |
| Systolic blood pressure per 10 mmHg |  |  | 0.7 (0.0, 1.4) |
| Fasting glucose per 10 mg/dL |  |  | -0.1 (-0.5, 0.3) |
| On hypertension medications |  |  | 1.1 (-1.3, 3.5) |
| On diabetes medications |  |  | 0.6 (-3.6, 4.7) |
| eGFR per 5 mL/min/1.73 m² |  |  | 0.1 (-0.2, 0.5) |
| LVH |  |  | -6.1 (-15, 2.9) |
| QT prolongation drugs (known+possible vs. conditional+none) |  |  | 0.0 (-3.5, 3.5) |
| Cocaine use |  |  | 3.5 (-0.3, 7.2) |
| **^§^**Models 2 and 3 further controlled for MACS enrollment site  ^ϯ^Mean T-wave onset to T-wave peak duration among men at the average and referent values of continuous and categorical covariates, respectively: i.e. white, HIV-uninfected men aged 57 years, with heart rate of 66 bpm, BMI of 27 kg/m^2^, SBP of 130 mmHg, fasting glucose level of 100 mg/dL, eGFR of 85 mL/min/1.73 m², who did not use hypertension/diabetes medication, opioids, or cocaine, did not use QT prolongation drugs or only used conditional QT prolongation drugs, had no smoking history, no LVH on ECG, alcohol use ≤13 drinks per week, and enrolled before 2001.  **P* ≤0.05; ***P* <0.01 | | | |

| **Supplemental Table 3**: Risk factors for T-wave peak to T-wave end duration among all 1426 participants, in univariate (model 1), partially adjusted (model 2) and fully adjusted (model 3) analyses. | | | |
| --- | --- | --- | --- |
|  | **Model 1** | **Model 2^§^** | **Model 3^§^** |
|  | Mean difference  (95% CI) | Mean difference  (95% CI) | Mean difference  (95% CI) |
| Intercept^ϯ^ | **98.3 (97.4, 99.2)**** | **97.5 (95.8, 99.1)**** | **97.1 (95.4, 98.9)**** |
| HIV infected (vs. uninfected) | 0.9 (-0.3, 2.2) | **1.4 (0.1, 2.7)*** | **1.6 (0.3, 2.9)*** |
| Age per 5 years |  | **0.4 (0.1, 0.8)*** | **0.4 (0.1, 0.8)*** |
| Race |  |  |  |
| Black (vs Caucasian) |  | **2.0 (0.4, 3.7)*** | **2.1 (0.4, 3.7)*** |
| Hispanic/Other (vs Caucasian) |  | 1.9 (-0.2, 4.0) | 1.9 (-0.1, 3.9) |
| Enrolled after 2001 |  | 0.3 (-1.5, 2.0) | 0.1 (-1.6, 1.9) |
| Heart rate per 5 bpm |  | **-1.1 (-1.3, -0.8)**** | **-1.1 (-1.4, -0.8)**** |
| Body mass index per 5 kg/m^2^ |  |  | **1.5 (0.9, 2.1)**** |
| Smoking (per 10 cumulative pack-years) |  |  | **-0.5 (-0.8, -0.2)**** |
| Alcohol use >13 drinks per week |  |  | -0.8 (-3.0, 1.4) |
| Opioid use |  |  | 1.0 (-1.3, 3.4) |
| Systolic blood pressure per 10 mmHg |  |  | 0.0 (-0.4, 0.4) |
| Fasting glucose per 10 mg/dL |  |  | 0.0 (-0.3, 0.2) |
| On hypertension medications |  |  | 0.1 (-1.3, 1.5) |
| On diabetes medications |  |  | -2.3 (-4.7, 0.1) |
| eGFR per 5 mL/min/1.73 m² |  |  | **-0.2 (-0.4, 0.0)*** |
| LVH on ECG (Cornell voltage) |  |  | **12.5 (7.3, 17.8)**** |
| QT prolongation drugs (known + possible vs. conditional + none) |  |  | 1.2 (-0.8, 3.3) |
| Cocaine use |  |  | 0.2 (-2.0, 2.4) |

**^§^**Models 2 and 3 further adjusted for MACS enrollment site (data not shown).

^ϯ^Mean T-wave peak to T-wave end duration among men at the average and referent values of continuous and categorical covariates, respectively: i.e. white, HIV-uninfected men aged 57 years, with heart rate of 66 bpm, BMI of 27 kg/m^2^, SBP of 130 mmHg, fasting glucose level of 100 mg/dL, eGFR of 85 mL/min/1.73 m², who did not use hypertension/diabetes medication, opioids, or cocaine, did not use QT prolongation drugs or only used conditional QT prolongation drugs, had no smoking history, no LVH on ECG, alcohol use ≤13 drinks per week, and enrolled before 2001.

**P* ≤0.05; ***P* <0.01

| **Supplemental Table 4:** HIV-specific risk factors for longer T-wave peak to T-wave end component among 774 HIV+ participants**^§^** | | |
| --- | --- | --- |
|  | Mean difference in T-onset to T-peak  (msec, 95% CI) | Mean difference in T-peak to T-end  (msec, 95% CI) |
| Duration of HAART (years) | 0.2 (-0.1,0.4) | -0.1 (-0.2, 0.1) |
| Nadir CD4+ T-cell count <500 cells/mm3 | -1.7 (-5.2,1.7) | -0.8 (-2.9, 1.3) |
| Current CD4+ T-cell count <500 cells/mm3 | 1.3 (-2,4.6) | -1.5 (-3.5, 0.5) |
| Undetectable HIV RNA viral load (<50 copies/mL) | 0.5 (-3.3,4.3) | **-2.9 (-5.2, -0.7)*** |
| History of AIDS | 1.4 (-3.5,6.3) | -1.4 (-4.3, 1.6) |
| On protease inhibitors (PI) | **-3.3 (-6.5,0)*** | **3.1 (1.1, 5.0)**** |
| Cumulative years of PI use | 0.0 (-0.2,0.2) | 0.0 (-0.1, 0.2) |
| On efavirenz | 0.6 (-8.8,10) | -1.4 (-7.0, 4.2) |
| Cumulative years of efavirenz use | 0.1 (-0.2,0.3) | -0.1 (-0.3, 0.0) |
| On rilpivirine | 8.9 (-5.5,23.2) | -1.1 (-9.6, 7.5) |
| Cumulative years of rilpivirine use | -0.5 (-1.9,0.8) | -0.1 (-0.9, 0.7) |

**^§^**Each HIV factor was assessed in a separate model with adjustment for age per 5 years, race, MACS site, wave of MACS enrollment (before/after 2001), heart rate per 5 bpm, BMI, cumulative pack-year of smoking, heavy alcohol use >13 drinks/week, systolic blood pressure, receipt of medications to treat hypertension or diabetes, opioid use, cocaine use, fasting glucose level, eGFR per 5 mL/min/1.73 m², ECG LVH, and use of QT prolongation drugs (known+possible vs. conditional+none).

**P* ≤0.05; ***P* <0.01
